# Agrochemical synergism imposes higher risk to Neotropical bees than to honeybees

**DOI:** 10.1098/rsos.160866

**Published:** 2017-01-18

**Authors:** Hudson V. V. Tomé, Gabryele S. Ramos, Micaele F. Araújo, Weyder C. Santana, Gil R. Santos, Raul Narciso C. Guedes, Carlos D. Maciel, Philip L. Newland, Eugênio E. Oliveira

**Affiliations:** 1 Departamento de Entomologia, Universidade Federal de Viçosa, 36570-900 Viçosa-MG, Brazil; 2 EAG Laboratories, 13709 Progress Boulevard no. 24, Suite S163, Alachua, FL, 32615USA; 3 Programa de Pós-Graduação em Produção Vegetal, Universidade Federal do Tocantins, Gurupi, TO 77410-530, Brazil; 4 Department of Electrical Engineering, São Carlos School of Engineering, University of São Paulo, São Carlos, SP, Brazil; 5 Biological Sciences, University of Southampton, Highfield Campus, Southampton SO17 1BJ, UK

**Keywords:** synergy, stressors, pesticide impacts, honeybee, stingless bee, pollinators

## Abstract

Bees are key pollinators whose population numbers are declining, in part, owing to the effects of different stressors such as insecticides and fungicides. We have analysed the susceptibility of the Africanized honeybee, *Apis mellifera*, and the stingless bee, *Partamona helleri,* to commercial formulations of the insecticides deltamethrin and imidacloprid. The toxicity of fungicides based on thiophanate-methyl and chlorothalonil were investigated individually and in combination, and with the insecticides. Results showed that stingless bees were more susceptible to insecticides than honeybees. The commercial fungicides thiophanate-methyl or chlorothalonil caused low mortality, regardless of concentration; however, their combination was as toxic as imidacloprid to both species, and over 400-fold more toxic than deltamethrin for *A. mellifera*. There were highly synergistic effects on mortality caused by interactions in the mixture of imidacloprid and the fungicides thiophanate-methyl, chlorothalonil and the combined fungicide formulation in *A. mellifera,* and also to a lesser extent in *P. helleri*. By contrast, mixtures of the deltamethrin and the combined fungicide formulation induced high synergy in *P. helleri*, but had little effect on the mortality of *A. mellifera*. Differences in physiology and modes of action of agrochemicals are discussed as key factors underlying the differences in susceptibility to agrochemicals.

## 1. Introduction

Bees are key pollinators that provide ecosystem services to wild and economically cultivated plants in temperate and tropical regions around the planet [1,2]. Their population numbers, however, are threatened owing to multiple stressors that include habitat fragmentation, pathogens, parasites, poor nutrition and pesticides [3–6]. Recent studies have shown that pesticides alone, or in combination with other stressors, contribute to colony losses [7–9], and it is the impact of simultaneous multiple stressors that is thought to have the greatest impact on bees. Insecticides, such as pyrethroids and systemic neonicotinoids, have been widely studied in bumblebees and European honeybees [7,9–11], yet the impact of multiple agrochemicals (i.e. insecticides, herbicides, fungicides and even leaf fertilizers) on other pollinators, or other species of bees that occupy Neotropical regions, such as Africanized honeybees and stingless bees [12–18], has yet to be determined.

Agrochemicals sprayed on many managed crops are an important threat to survival of bees, and a wide range of compounds and their metabolites have been identified inside colonies [19,20]. Although there is no evidence that one chemical alone is solely responsible for colony losses, the combination of pesticide residues may be dangerous for bees. Insecticides and fungicides that are used in agricultural environments, mainly during the blooming season, can affect directly forager bees and contaminate pollen and nectar brought to the colonies [19,21]. Although fungicides are considered safe for bees, synergistic interactions between active ingredients or adjuvants integrated into their formulations may increase risks to pollinators [11,22,23], especially when farmers routinely use tank mixtures of insecticides and fungicides on crops to reduce spraying costs associated with pest management. In synergistic interactions, the biological activity of a mixture is greater than the sum of expected individual responses to each chemical [24]. Despite the scarcity of studies focusing on the synergy between agrochemicals, and pesticides in particular, on pollinators in general, mixtures of insecticides and ergosterol biosynthesis inhibitor (EBI) fungicides have been shown to lead to higher toxicity in bees [11,25,26].

Here, we have evaluated the susceptibility of Africanized honeybees, *Apis mellifera*, and the stingless bee species *Partamona helleri.* Both species are common pollinators of melon and watermelon in the Neotropics and thus frequently exposed to commercial formulations of insecticides (e.g. deltamethrin and imidacloprid) and non-EBI fungicides (e.g. thiophanate-methyl and chlorothalonil) [6,15,18]. We demonstrate that the synergistic effects of agrochemicals pose a higher risk to *P. helleri* compared with *A. mellifera*.

## 2. Material and methods

### 2.1. Insects and pesticides

Colonies of the stingless bee *P. helleri* and the Africanized honeybee *A. mellifera* established in an experimental apiary at the Federal University of Viçosa (UFV, Viçosa, MG, Brazil, 20°45′ S, 42°52′ W) were used in all experiments. Four to six bee colonies of each species were used in these experiments. Forager bees collected from each colony were transferred to the laboratory and fasted for 1 h under controlled temperatures, as found in their respective colonies (*P. helleri*: 28 ± 1°C. *A. mellifera*: 34 ± 1°C) and with relative humidity of 70 ± 10% in complete darkness prior to the bioassays. The fasting period before pesticide exposure was necessary to standardize diet consumption for the tested bees. We used commercial pesticide formulations at their respective label rates to allow realistic estimates aimed at risk assessment. The pesticides used included the pyrethroid deltamethrin (Decis; 25 g of active ingredients (a.i.) l^−1^, Bayer CropScience, São Paulo, SP, Brazil) and the neonicotinoid imidacloprid (Evidence; 700 g a.i. l^−1^; Bayer CropScience). We also used commercial formulations of fungicides that do not inhibit the synthesis of ergosterol (non-EBI fungicides), namely chlorothalonil (Dacobin; 750 g a.i. kg^−1^, Iharabras, São Paulo, SP, Brazil) and thiophanate-methyl (Cercobin; 700 g a.i. kg^−1^, Iharabras). These non-EBI fungicides were used alone or mixed in a commercial formulation containing 200 g of thiophanate-methyl kg^−1^ and 500 g of chlorothalonil kg^−1^ (Cerconil, Iharabras). Furthermore, as mixtures of deltamethrin or imidacloprid with these fungicides are frequently used in melon production fields pollinated by both *A. mellifera* and *P. helleri* in Neotropical regions, we also mixed the insecticides (i.e. deltamethrin or imidacloprid) with these fungicides, where the fungicides had a fixed concentration of 10 g l^−1^.

### 2.2. Concentration–mortality bioassays

Concentration–mortality bioassays were performed by orally exposing forager bees of each species to different concentrations of insecticides alone or mixed with fungicides. Five to seven concentrations of each pesticide were used to estimate concentration–mortality curves. The pesticides were diluted in honey-based syrup solution (50%, v/v) and offered to bees in 2 ml Eppendorf tubes inserted into low-density plastic containers (250 ml). Each plastic container was used as an experimental unit containing 20 forager bees fed on 1 ml of pesticide-contaminated honey solution (except for untreated bees, i.e. control). The bees were fasted for 1 h prior to allowing access to the pesticide-contaminated diet. After 5 h of oral pesticide exposure, the bees were provided with an insecticide-free diet ad libitum, and mortality was recorded 24 h after diet replacement. Bees were considered dead if unable to move when prodded with a fine hair brush. Each replicate consisted of a plastic container containing forager bees from the same colony, and three to six different colonies were used in the bioassays to account for intercolony variation in response.

### 2.3. Body mass and respirometry bioassays

The body mass and respiration rates of *A. mellifera* and *P. helleri* foragers were measured to determine whether differences could be related to the susceptibility of the two species to pesticides. Bees were maintained for 1 h of fasting before weighing to avoid variations in weight owing to prior feeding. Twenty unexposed bees of each species (i.e. four per colony) were weighed on an electronic analytical balance (model XS3DU, Mettler Toledo, Columbus, OH), and another 20 bees were used for respirometry bioassays. CO_2_ production was recorded using a TR3C respirometer equipped with a CO_2_ analyser (Sable Systems International, Las Vegas, NV). Each forager bee was maintained individually in 25 ml glass chambers in a completely closed system. CO_2_ production (µl CO_2_ h^−1^ bee^−1^) was determined after a 2 h period by injecting CO_2_-free air into the chamber for 2 min at a flow rate of 600 ml min^−1^. An air current directed the CO_2_ produced by the bees to an infrared reader connected to the system. CO_2_ production was also determined in a control chamber without an insect. Twenty bees of each species (i.e. four per colony) unexposed to insecticide or fungicide were analysed.

### 2.4. Statistical analysis

Concentration–mortality curves were estimated by probit analyses, using the PROC PROBIT procedure (SAS Institute 2008). The differential insecticide susceptibility between *A. mellifera* and *P. helleri* was calculated for each pesticide based on the estimated LC_50_ (i.e. the lethal concentration capable of killing 50% of tested bees) for each insecticide and bee species, and the susceptibility ratios (SR_50_) were estimated by dividing the LC_50_ value obtained for *A. mellifera* by the LC_50_ value obtained for *P. helleri* [27]. The 95% confidence limits of these toxicity rate estimates were considered to be significantly different (*p*  <  0.05) if they did not include the value 1 [27]. The suitability of the probit model to estimate the pesticide toxicity was based on the low *χ*^2^-values (less than 6.6) and high *p*-values (greater than 0.05). Results obtained for body mass and respiration rates were analysed using non-parametric analyses of variance (ANOVA) on ranks (Kruskal–Wallis test), because normality and homoscedasticity assumptions of analysis of variance were not satisfied (Sigmaplot v. 12.5; Systat, San Jose, CA).

## 3. Results

### 3.1. Susceptibility to isolated insecticides and fungicides

**Figure 1.**
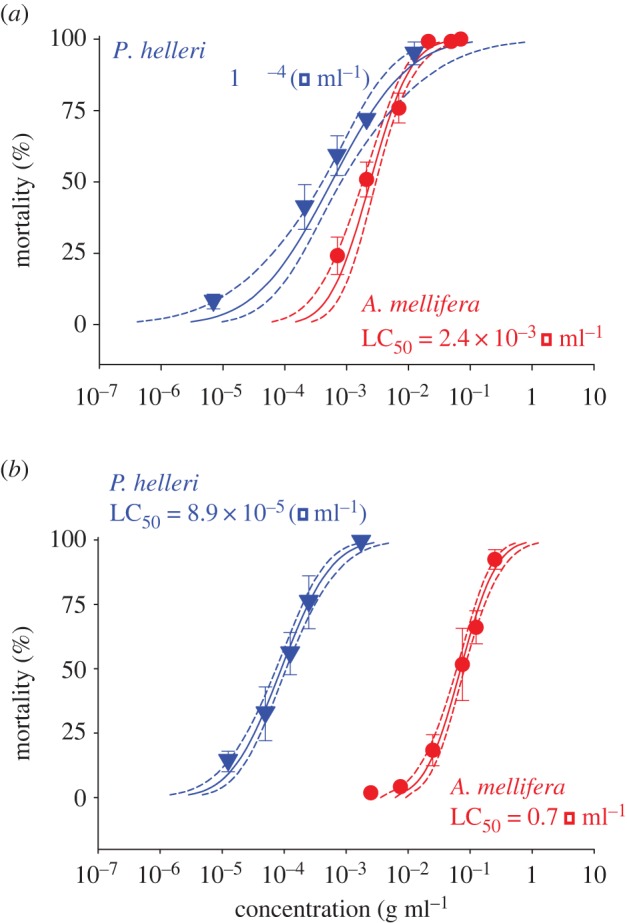
Impact of insecticides on bee mortality. Concentration–mortality curves of the forager bees of *Apis mellifera* and *Partamona helleri* exposed to imidacloprid (*a*) and deltamethrin (*b*). LC_50_ values are given and dotted lines represent the 95% fiducial limits of each curve. Symbols represent concentrations tested (on axis *x*) and observed mortality (on axis *y*). Vertical bars represent standard error (s.e.) of the mean.

The effects of insecticide concentration on mortality were determined for both *P. helleri* and *A. mellifera* for imidacloprid and deltamethrin (table 1 and figure 1). Although basing our estimates in concentration rather than in dose, we also estimated the respective doses (in ng of active ingredient bee^−1^) and provide them in table 2 as a general reference for extrapolation. These estimations were based on the food consumption by each bee species, achieved by weighing the feeders prior to and after 5 h of exposure to diet. The average of the consumption was 0.026 µl to *A. mellifera* and 0.016 µl to *P. helleri*.

**Table 1.** Oral relative toxicity (in µg a.i. ml^−1^**)** of pesticides to *A. mellifera* and *P. helleri.*

				LC_50_ (95% FL)			*SR*^b^ LC_50_
insecticides	species	*n*	slope ± s.e.	µg a.i. ml^−1^	*χ*^2^	*P*^a^	(95% FL)
deltamethrin	*A. mellifera*	652	0.3 ± 0.1	7.1 × 10^5^ (6.1 × 10^5^ to 8.3 × 10^5^)	2.7	0.60	—
	*P. helleri*	588	4.7 ± 0.4	891.1 (739.3–998.2)	3.9	0.26	8.0 × 10^2^ (2.9 × 10^2^ to 2.2 × 10^3^)^c^
imidacloprid	*A. mellifera*	1080	5.0 ± 0.4	240.1 (184.2–292.3)	6.6	0.18	—
	*P. helleri*	522	3.3 ± 0.5	573.3 (402.4–807.1)	2.8	0.41	4.2 (2.1–8.4)^c^
Cerconil (thiophanate-methyl + chlorothalonil)	*A. mellifera*	601	3.6 ± 0.3	1498.1 (1208.3 –1894.5)	2.6	0.45	—
	*P. helleri*	740	6.2 ± 0.3	990.2 (810.3 –1182.3)	5.9	0.20	1.6 (1.0–5.5)
Cerconil + imidacloprid	*A. mellifera*	840	3.3 ± 0.3	1.5 (0.7–2.8)	1.5	0.67	—
	*P. helleri*	588	1.6 ± 0.3	2.9 (0.9 –19.8)	3.6	0.16	0.5 (0.3–7.3)
thiophanate-methyl + imidacloprid	*A. mellifera*	500	3.0 ± 0.6	19.1 (9.4 –36.2)	2.7	0.43	—
	*P. helleri*	480	1.9 ± 0.4	29.3 (12.3–91.4)	4.9	0.18	0.6 (0.1–3.5)
chlorothalonil + imidacloprid	*A. mellifera*	600	5.4 ± 0.7	98.9 (65.0 –138.4)	4.4	0.22	—
	*P. helleri*	480	2.9 ± 0.4	6.8 (3.3 –12.8)	1.5	0.46	14.6 (4.1–55.1)^c^
Cerconil + deltamethrin	*A. mellifera*	840	1.2 ± 0.1	2.1 × 10^6^ (1.8 × 10^6^ to 2.4 × 10^6^)	7.4	0.19	—
	*P. helleri*	478	4.6 ± 0.4	4.9 (3.4 –6.9)	5.8	0.21	4.3 × 10^4^ (2.4 × 10^4^ to 7.8 × 10^4^)^c^
thiophanate-methyl + deltamethrin	*A. mellifera*	480	1.7 ± 0.2	2.3 × 10^6^ (1.8 × 10^6^ to 2.7 × 10^6^)	2.9	0.23	—
	*P. helleri*	480	6.9 ± 0.8	1591.9 (1208.3 –2106.1)	1.3	0.72	1.4 × 10^2^ (8.6 × 10^1^ to 2.4 × 10^2^)^c^
chlorothalonil + deltamethrin	*A. mellifera*	596	1.7 ± 0.1	3.1 × 10^6^ (2.8 × 10^6^ to 3.4 × 10^6^)	0.9	0.81	—
	*P. helleri*	480	5.8 ± 0.7	1.6 (1.2 –2.2)	4.6	0.20	2.0 × 10^2^ (1.3 × 10^2^ to 3.1 × 10^2^)^c^

^a^Probability values.

^b^Susceptibility ratio (LC_50_ to *A. mellifera*/LC_50_ to *P. helleri*).

^c^indicate when the SRs are significantly different. (i.e. the 95% CL of SR did not include the value 1).

**Table 2.** Lethal doses of pesticides (in ng a.i. bee^−1^) to *A. mellifera* and *P. helleri* under oral exposure.

insecticides	species	LD_50_^a^ (95% FL) (ng a.i. bee^−1^)
deltamethrin	*A. mellifera*	18.4 (15.7–21.0)
	*P. helleri*	0.014 (0.011–0.016)
imidacloprid	*A. mellifera*	0.063 (0.047–0.076)
	*P. helleri*	0.091 (0.064–0.012)
Cerconil (thiophanate-methyl + chlorothalonil)	*A. mellifera*	0.039 (0.031–0.050)
	*P. helleri*	0.015 (0.012–0.019)
Cerconil + imidacloprid	*A. mellifera*	3.9 × 10^−5^ (1.7 × 10^−5^ to 7.3 × 10^−5^)
	*P. helleri*	4.6 × 10^−5^ (1.5 × 10^−5^ to 1.6 × 10^−4^)
thiophanate-methyl + imidacloprid	*A. mellifera*	5.0 × 10^−4^ (2.4 × 10^−4^ to 9.4 × 10^−4^)
	*P. helleri*	4.6 × 10^−4^ (1.9 × 10^−4^ to 1.4 × 10^−3^)
chlorothalonil + imidacloprid	*A. mellifera*	2.6 × 10^−3^ (1.7 × 10^−3^ to 3.6 × 10^−3^)
	*P. helleri*	1.0 × 10^−4^ (5.2 × 10^−5^ to 2.0 × 10^−4^)
Cerconil + deltamethrin	*A. mellifera*	5.5 (4.7–6.3)
	*P. helleri*	7.8 × 10^−5^ (5.4 × 10^−5^ to 1.1 × 10^−4^)
thiophanate-methyl + deltamethrin	*A. mellifera*	6.0 (4.7–7.1)
	*P. helleri*	0.025 (0.019–0.032)
chlorothalonil + deltamethrin	*A. mellifera*	7.8 (7.7–8.9)
	*P. helleri*	0.025 (0.019–0.035)

^a^Estimated LD_50_ considering the average consumption of 0.026 µl to *A. mellifera* and 0.016 µl to *P. helleri*.

With increasing concentrations of imidacloprid (in the range of 0.4 to 8.0 × 10^5^ µg a.i. ml^−1^) mortality increased in both *P. helleri* and *A. mellifera*, from less than 10% to 100%. The LC_50_ for *P. helleri* was 573.3 µg a.i. ml^−1^, whereas for *A. mellifera* it was 240.1 µg a.i. ml^−1^. Based on the LC_50_ measures obtained from these concentration–mortality bioassays, *P. helleri* was more susceptible to imidacloprid than *A. mellifera* (SR_50_ = 4.2 [2.1–8.4]-fold). Increasing concentrations of deltamethrin (in the range of 25 µg a.i. ml^−1^ to 2.5 × 10^6^ µg a.i. ml^−1^) again increased mortality for both *P. helleri* and *A. mellifera*. The LC_50_ for *P. helleri* was 891.1 µg a.i. ml^−1^, whereas for *A. mellifera* it was 7.1 × 10^5^ µg a.i. ml^−1^. Based on these LC_50_ measures, *P. helleri* was more susceptible to deltamethrin than *A. mellifera* (SR_50_ = 800 [290–2200]-fold; table 1 and figure 1).

**Figure 2.**
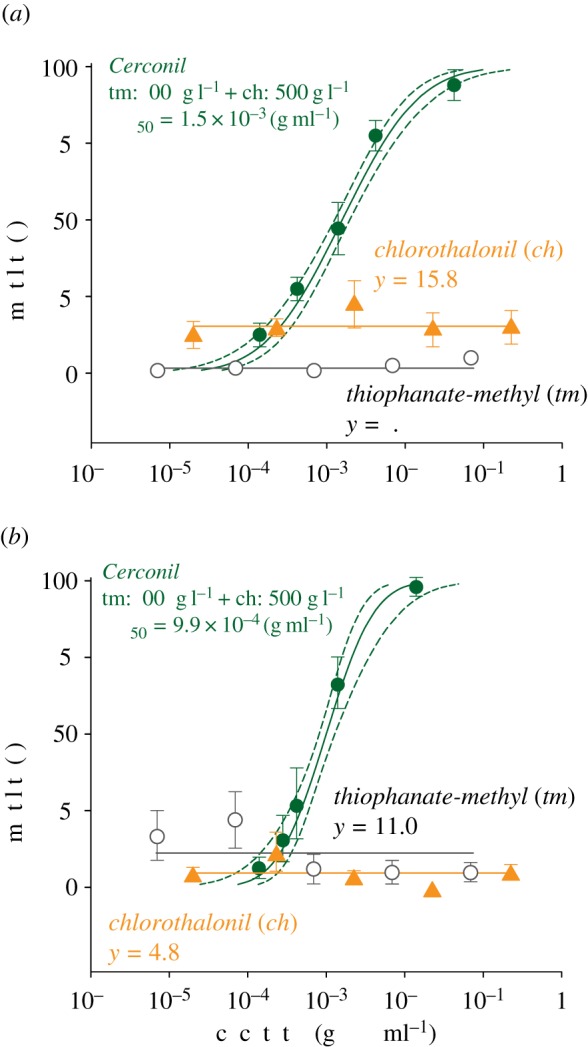
Impact of fungicides on bee mortality. Concentration–mortality curves of the forager bees of *Apis mellifera* (*a*) and *Partamona helleri* (*b*) exposed to the fungicides chlorothalonil (ch), thiophanate-methyl (tm) and Cerconil (mixture of chlorothalonil and thiophanate-methyl). LC_50_ values are indicated, and dotted lines represent the 95% fiducial limits of each curve. Symbols represent concentrations tested (on *x-* axis) and observed mortality (on *y-*axis). Vertical bars represent standard error (s.e.) of the mean.

The effects of the fungicide formulation Cerconil and its two active ingredients chlorothalonil and thiophanate-methyl were also analysed. There was no concentration-dependent effect on mortality to the two active components applied alone for either bee species with mortality less than 25% for thiophanate-methyl in both *A. mellifera* (figure 2*a*) and *P. helleri* (figure 2*b*). Mortality was also independent of concentration for chlorothalonil for both species and again less than 25% (figure 2*a*,*b*).

By contrast, when bees were fed on food contaminated with a mixture of both fungicide active ingredients (i.e. Cerconil formulation), mortality in both *A. mellifera* (figure 2*a*) and *P. helleri* (figure 2*b*) increased markedly with increased fungicide concentration (table 1), allowing the estimation of the concentration–mortality curves for both bee species (table 1 and figure 2). For *A. mellifera*, the LC_50_ was 1498.1 µg of Cerconil ml^−1^, whereas for *P. helleri* the LC_50_ was 990.2 µg of Cerconil ml^−1^.

The synergistic actions of these non-EBI fungicides led to greater mortality levels of both bee species compared with the mortality caused by imidacloprid or by deltamethrin (compare figures 1 and 2). Based on susceptibility ratios both *A. mellifera* (SR_50_ = 1.5 [0.9–1.9]-fold) and *P. helleri* (SR_50_ = 0.6 [0.3–1.1]-fold) were as susceptible to the combined fungicides (Cerconil) as to imidacloprid. Similar susceptibility levels were observed when comparing deltamethrin and Cerconil for *P. helleri* (SR_50_ = 0.9 [0.8–1.2]-fold), but *A. mellifera* was more than 400-fold more susceptible to Cerconil than to deltamethrin.

### 3.2. Non-ergosterol biosynthesis inhibitor fungicides potentiate the insecticide actions on both *Apis mellifera* and *Partamona helleri*

**Figure 3.**
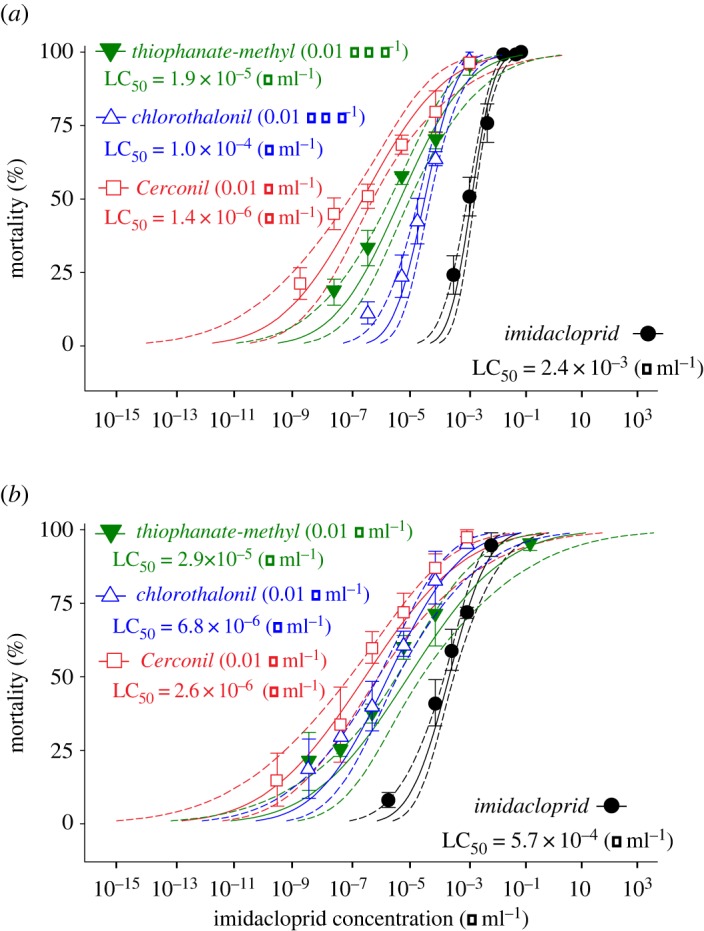
Effects of combined exposure to the neonicotinoid imidacloprid and fungicides on bee mortality. Concentration–mortality curves of *Apis mellifera* (*a*) and *Partamona helleri* (*b*) exposed to mixtures of imidacloprid and the fungicides chlorothalonil (ch), thiophanate-methyl (tm) and Cerconil (mixture of chlorothalonil and thiophanate-methyl). LC_50_ values are indicated and dotted lines represent the 95% fiducial limits of each curve. Symbols represent concentrations tested (on *x-*axis) and observed mortality (on *y-*axis). Vertical bars represent standard error (s.e.) of the mean.

Mixtures of the non-EBI fungicide, Cerconil, and each of its active ingredients (i.e. thiophanate-methyl and chlorothalonil) with the insecticide imidacloprid significantly increased mortality of *A. mellifera* (figure 3*a*) compared with when imidacloprid was used alone. A mixture of chlorothalonil (0.01 g a.i. ml^−1^) with a range of imidacloprid concentrations was 23.9 (13.7–41.8)-fold more toxic to *A. mellifera* than imidacloprid alone (figure 3*a*). Similarly, a mixture of thiophanate-methyl (0.01 g a.i. ml^−1^) with imidacloprid potentiated the effect of imidacloprid alone on *A. mellifera* by approximately 126.0 (80.6–191.0)-fold (figure 3*a*). When imidacloprid was mixed with the fungicide formulation containing both non-EBI active ingredients (Cerconil), the mortality of *A. mellifera* was 1589.8 (1035.7–2769.2)-fold higher than that observed for imidacloprid alone (figure 3*a*). In *P. helleri*, mixtures of the non-EBI fungicides with imidacloprid also potentiated the effects of imidacloprid (SR_50_ for imidacloprid + chlorothalonil = 83.4 [42.7–181.4]; SR_50_ for imidacloprid + thiophanate-methyl = 19.3 [13.3–88.9] and SR_50_ for imidacloprid + chlorothalonil + thiophanate-methyl = 197.0 [100.6–430.1]; figure 3*b*).

**Figure 4.**
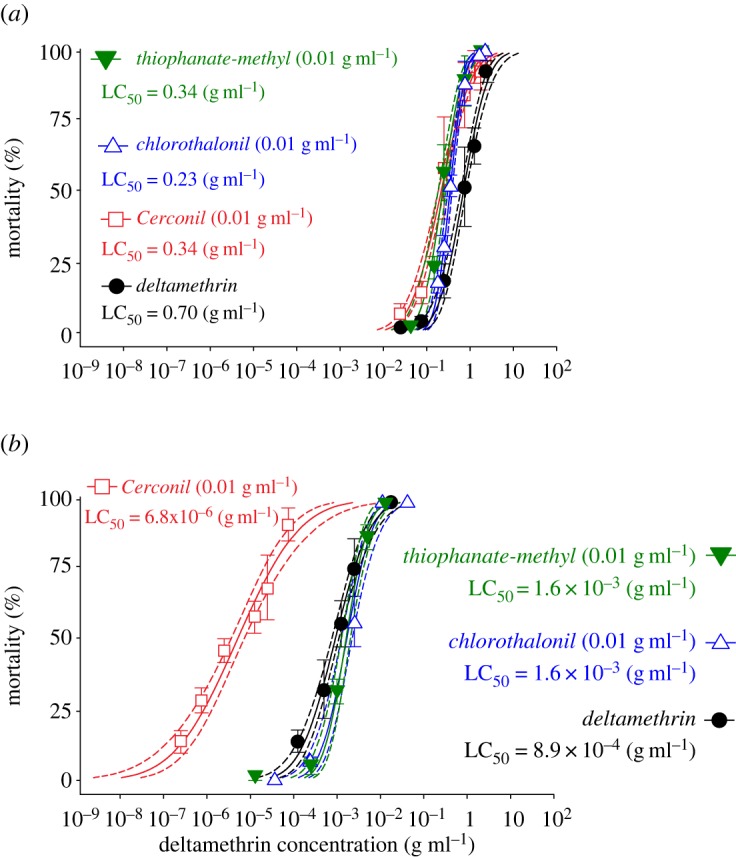
Effects of combined exposure to the pyrethroid deltamethrin and fungicides on bee mortality. Concentration–mortality curves of *Apis mellifera* (*a*) and *Partamona helleri* (*b*) exposed to mixtures of deltamethrin and the fungicides chlorothalonil (ch), thiophanate-methyl (tm) and Cerconil (mixture of chlorothalonil and thiophanate-methyl). LC_50_ values are indicated and dotted lines represent the 95% fiducial limits of each curve. Symbols represent concentrations tested (on *x-*axis) and observed mortality (on *y-*axis). Vertical bars represent standard error (s.e.) of the mean.

Mixtures of the non-EBI fungicides thiophanate-methyl and chlorothalonil, individually, with the insecticide deltamethrin led to small increases in mortality compared with deltamethrin alone in both species (figure 4). For example, mixtures of each of thiophanate-methyl or chlorothalonil with deltamethrin resulted in toxicity levels less than fourfold for both species (figure 4*a*,*b*). Mixtures of both non-EBI fungicide ingredients (Cerconil) together with deltamethrin caused a small increase in mortality of *A. mellifera* (figure 4*a*), but a 177.0 (58.4–536.7)-fold increase in *P. helleri* compared with deltamethrin alone (figure 4*b*). Thus, *P. helleri* was about 4300.4 [2200.1–7800.3]-fold more susceptible than *A. mellifera* to the mixture of deltamethrin and the fungicide formulation containing both non-EBI active ingredients (table 1 and figure 4*b*).

### 3.3. Body mass and respiration rates of *Apis mellifera* and *Partamona helleri*

**Figure 5.**
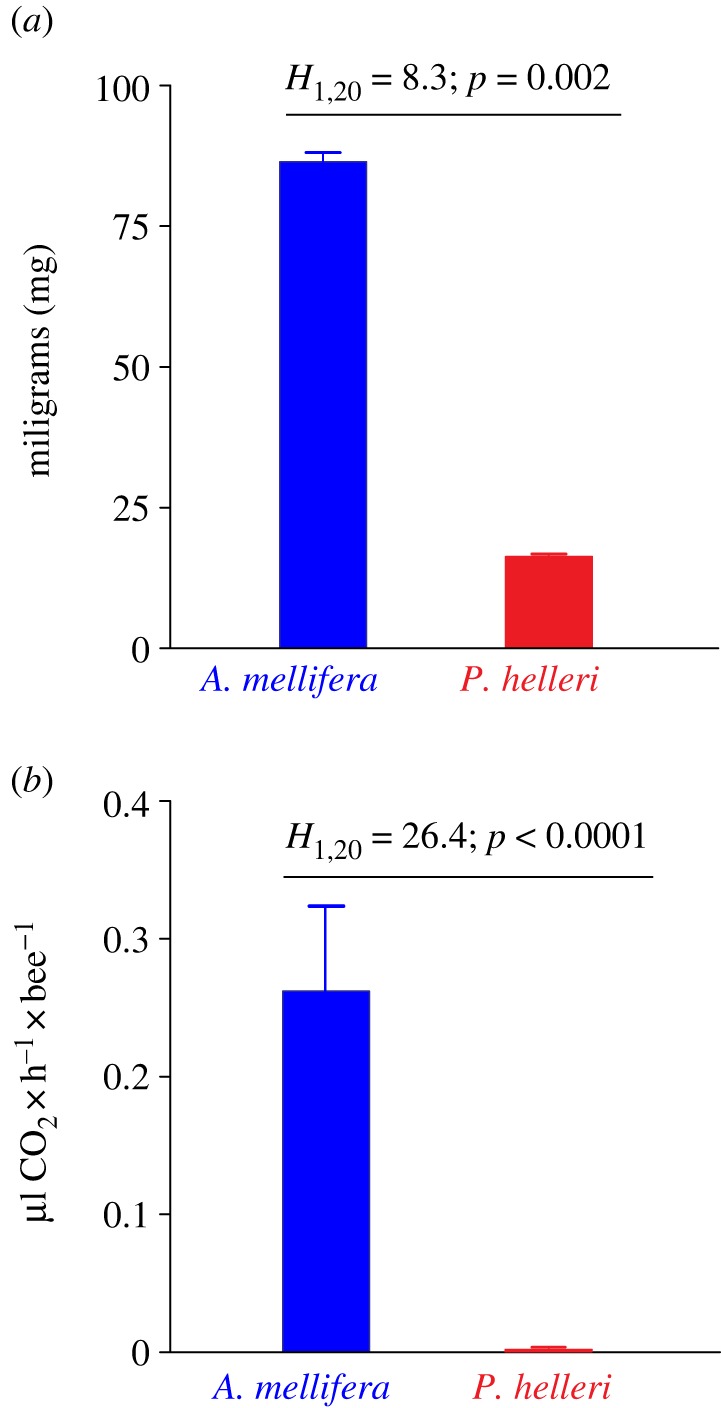
Body mass (*a*) and respiration rate (*b*) of unexposed individual (±s.e.) adult workers of the stingless bee *Partamona helleri* and honeybee *Apis mellifera*. Based on Kruskal–Wallis tests, there were significant differences between species for body mass (*H*_1,20_ = 8.3, *p* = 0.002) and respiration rates (*H*_1,16_ = 26.2, *p* < 0.001).

The body mass of *A. mellifera* was 86.4 ± 0.6 mg (mean ± s.e.), whereas that of *P. helleri* was 16.2 ± 0.02 mg (mean ± s.e.; figure 5*a*). A Kruskal–Wallis test showed that *A. mellifera* were statistically heavier than *P. helleri* (*H*_1,20_ = 8.3, *p* = 0.002). The respiration rates were significantly higher in *A. mellifera* (0.26 ± 0.01 µl CO_2_ h^−1^ bee^−1^) compared with *P. helleri* (0.002 ± 0.0003 µl CO_2_ h^−1^ bee^−1^; figure 5*b*). A Kruskal–Wallis test showed that respiration rates for the two species of bees were significantly different (*H*_1,20_ = 26.2, *p* < 0.001). While the weight difference between *A. mellifera* and *P. helleri* was approximately 5.3-fold, the respiration rate of *A. mellifera* was approximately 460-fold higher than that of *P. helleri*.

## 4. Discussion

During foraging, bee pollinators can be exposed to different agrochemicals, including insecticides, fungicides and herbicides in crop landscapes around the world. Here we demonstrate that both honeybees and Neotropical stingless bees were susceptible to mixtures of fungicides (i.e. chlorothalonil and thiophanate-methyl) that are normally considered safe in bee risk assessments on their own [28,29]. In addition, we show that Neotropical stingless bees were more susceptible to insecticides (e.g. deltamethrin and imidacloprid) compared with the honeybees. These findings reinforce the notion that *A. mellifera* is not a faithful model to assess the safety of agrochemicals when compared with other species of bees [18,30,31] and emphasize the limited value of extrapolating results of toxicity bioassays from honeybees to native pollinator species. Previous reports have shown that the combination of different chemicals in the environment can result in dangerous cocktails to pollinators [11,32]. However, the majority of studies reporting synergistic interactions between pesticides have focused largely on European honeybees neglecting Africanized honeybees and stingless bees that are routinely exposed to agrochemicals and their mixtures in Neotropical agricultural landscapes [11,18,22,24,33].

Fungicides are often sprayed on managed crops when bees are foraging and for this reason their residues, as well as those of other agrochemicals, have been detected in pollen in honeybee hives [19–21]. Although fungicides are considered safer to bees than other agrochemicals owing to their high LD_50_ values [28,29], some fungicide commercial formulations can increase insecticide toxicity [22,23,34]. Most of these studies, however, have focused on mixtures of insecticides such as pyrethroids or neonicotinoids and EBI fungicides (i.e. fungicides that inhibit synthesis of ergosterol) [11,25,26], which are known to alter the levels of cytochrome P_450_-mediated detoxification in honeybees, making them more susceptible to insecticides [33,35]. The effects of non-EBI fungicides such as thiophanate-methyl (an inhibitor of key enzymes of electron transport chain [36]) and chlorothalonil (a chloronitrile of broad spectrum with an unclear mode of action [37]) have received little attention.

We found that a commercial fungicide formulation, Cerconil, containing both chlorothalonil and thiophanate-methyl was as toxic to both bee species as imidacloprid, and at least 400-fold more toxic than the pyrethroid deltamethrin to *A. mellifera*. In addition, mixtures of deltamethrin or imidacloprid and the fungicides chlorothalonil, thiophanate-methyl or both fungicide ingredients (i.e. Cerconil) increased the mortality levels of pollinators with the magnitude of synergistic interactions depending on the type of insecticide in the mixture and the bee species. These findings may reflect agrochemical- or insect-related differences. For example, there is mounting evidence that commercial formulations of modern agrochemicals comprise components other than the active ingredients that elicit very different physiological effects on target and non-target organisms, usually enhancing the active ingredient activity [22,23,34]. Species-related differences may result from differences in life-history traits (e.g. sociality, body size, target-site sensitivity and capacity for detoxification by enhanced metabolism) of both bee species.

Although we know little of the detoxification processes in stingless bees, previous studies indicate that these bees are more susceptible to agrochemicals than other bees [18,30,31]. As respiration rate is affected by body mass and metabolic rate [38], differences in body mass and respiration rate were expected between both species [16,18]. Thus, the lower respiration rate of *P. helleri* is suggestive of a lower metabolic rate in this species compared with *A. mellifera,* which may be due to a lower capacity of xenobiotic detoxification of the former. However, other studies are necessary to fully investigate the relationship between detoxification process and metabolism in stingless bees.

Thus, although studies in field conditions are necessary to evaluate the real risk to which bees are subject when exposed to agrochemicals, our findings suggest that the mixtures of some non-EBI fungicides and insecticides routinely applied to crops pose higher risks to the native pollinators than to honeybees, compromising the recognized ecological and agricultural importance of the former bee species in Neotropical regions. Furthermore, our findings reinforce the notion that native bees might be more suitable models for agrochemical risk assessments in the Neotropical region as they are prevalent in these areas and are more susceptible to agrochemical exposure than honeybees.

## Supplementary Material

Suplementary material containing the raw data of the concentration-mortality bioassays. The pesticide effects on pollinator biodiversity is a global trend that is garnering much concern. Initially, there was significant concern about pesticide-mediated reductions in the number of honey bee pollinators, primarily Apis mellifera in the USA and in some European countries, but those initial concerns have been replaced by a broader concern related to the decline of pollinator bees in general. Here, we evaluated the impact of multiple stressors (e.g., insecticides and fungicides) on Africanized honey bees and stingless bees that are the most important pollinator insects in agriculture landscapes at the Neotropical regions. We demonstrated that the synergistic effects of agrochemicals (e.g., insecticides and fungicides) pose an increased risk to the Neotropical stingless bees Partamona helleri compared to the Africanized A. mellifera, which reinforce the notion that A. mellifera is not a faithful model to assess the safety of agrochemicals when compared with other species of bees and emphasize the limited value of extrapolating results of toxicity bioassays from honey bees to native pollinator species.

## Permission to carry out fieldwork

As we did not conduct any fieldwork in this study, no permissions for such issues were required.

## Data accessibility

All relevant data are within the paper and its electronic supplementary material files.
