# Supplementary material for: Agrochemical synergism imposes higher risk to Neotropical bees than to honeybees
Source: R Soc Open Sci. 2017 Jan 18;4(1):160866. doi: 10.1098/rsos.160866 (PMC5319351; doi:10.1098/rsos.160866)
Supplement: Suplementary material containing the raw data of the concentration-mortality bioassays. The pesticide effects on pollinator biodiversity is a global trend that is garnering much concern. Initially, there was significant concern about pesticide-mediated reductions in the number of honey bee pollinato [file rsos160866supp1.pdf]

Raw data used in the manuscript "Agrochemical synergism imposes higher risk to Neotropical bees than to honey bees" - by Tomé et al.

A. mellifera - Deltamethrin

Concentration (g/mL)	colony	total	dead
control	1	20	0
control	2	10	0
control	3	20	0
control	4	20	1
control	5	20	1
control	6	20	0
0.025	1	17	1
0.025	2	10	0
0.025	3	20	1
0.025	4	20	0
0.025	5	20	0
0.025	6	20	0
0.075	1	20	1
0.075	2	10	1
0.075	3	20	0
0.075	4	20	1
0.075	5	20	0
0.075	6	20	1
0.25	1	20	1
0.25	2	10	2
0.25	3	20	9
0.25	4	20	3
0.25	5	20	4
0.25	6	20	1
0.75	1	20	20
0.75	2	10	2
0.75	3	20	8
0.75	4	20	15
0.75	5	20	2
0.75	6	20	13
1.25	1	15	13
1.25	2	10	6
1.25	3	20	14
1.25	4	20	16
1.25	5	20	9
1.25	6	20	11
2.50	1	20	19
2.50	2	10	10
2.50	3	20	19
2.50	4	20	18
2.50	5	20	15
2.50	6	20	20

P. helleri - Deltamethrin

Concentração (g/mL)	colony	total	dead
**control**	**1**	20	0
**control**	**2**	20	2
**control**	**3**	20	0
**control**	**4**	20	1
**control**	**5**	20	8
**control**	**6**	20	1
**0.000125**	**1**	20	1
**0.000125**	**2**	20	1
**0.000125**	**3**	20	4
**0.000125**	**4**	20	5
**0.000125**	**5**	20	7
**0.000125**	**6**	20	3
**0.00025**	**1**	20	9
**0.00025**	**2**	20	20
**0.00025**	**3**	20	11
**0.00025**	**4**	20	3
**0.00025**	**5**	20	19
**0.00025**	**6**	20	19
**0.0005**	**1**	20	3
**0.0005**	**2**	20	5
**0.0005**	**3**	20	1
**0.0005**	**4**	20	5
**0.0005**	**5**	20	15
**0.0005**	**6**	20	5
**0.00125**	**1**	20	1
**0.00125**	**2**	20	1
**0.00125**	**3**	20	4
**0.00125**	**4**	20	5
**0.00125**	**5**	20	7
**0.00125**	**6**	20	3
**0.0025**	**1**	20	9
**0.0025**	**2**	20	20
**0.0025**	**3**	20	11
**0.0025**	**4**	20	3
**0.0025**	**5**	20	19
**0.0025**	**6**	20	20
**0.0175**	**1**	20	20
**0.0175**	**2**	20	19
**0.0175**	**3**	27	27
**0.0175**	**4**	21	21
**0.0175**	**5**	20	20
**0.0175**	**6**	20	20

A. mellifera -Imidacloprid

Concentration (g/mL)	Colony	total	dead
control	1	20	1
control	2	20	0
control	3	20	0
control	4	20	2
control	5	20	2
control	6	20	0
0.0000021	1	20	3
0.0000021	2	20	2
0.0000021	3	20	2
0.0000021	4	20	0
0.0000021	5	20	0
0.0000021	6	20	0
0.000021	1	20	6
0.000021	2	20	3
0.000021	3	20	3
0.000021	4	20	0
0.000021	5	20	0
0.000021	6	20	0
0.00021	1	20	1
0.00021	2	20	5
0.00021	3	20	7
0.00021	4	20	0
0.00021	5	20	0
0.00021	6	20	0
0.0021	1	20	10
0.0021	2	20	8
0.0021	3	20	8
0.0021	4	20	16
0.0021	5	20	10
0.0021	6	20	9
0.007	1	20	16
0.007	2	20	16
0.007	3	20	10
0.007	4	20	17
0.007	5	20	16
0.007	6	20	16
0.021	1	20	20
0.021	2	20	20
0.021	3	20	20
0.021	4	20	19
0.021	5	20	20
0.021	6	20	20
0.049	1	20	20
0.049	2	20	20
0.049	3	20	19
0.049	4	20	20

**0.049**	**5**	20	20
**0.049**	**6**	20	20
**0.07**	**1**	20	20
**0.07**	**2**	20	20
**0.07**	**3**	20	20
**0.07**	**4**	20	20
**0.07**	**5**	20	20
**0.07**	**6**	20	20
**0.21**	**1**	20	20
**0.21**	**2**	20	20
**0.21**	**3**	20	20
**0.21**	**4**	20	20
**0.21**	**5**	20	20
**0.21**	**6**	20	20

P. helleri **-Imidacloprid**

**Concentration (g/mL)**	**Colony**	**total**	**dead**
**control**	1	20	0
**control**	2	20	2
**control**	3	20	0
**control**	4	20	1
**control**	5	20	8
**control**	6	20	1
0.00000021	1	20	2
0.00000021	2	20	2
0.00000021	3	20	1
0.00000021	4	20	3
0.00000021	5	20	10
0.00000021	6	20	1
0.000007	1	20	1
0.000007	2	20	2
0.000007	3	20	3
0.000007	4	20	0
0.000007	5	20	17
0.000007	6	20	2
0.000021	1	20	9
0.000021	2	20	3
0.000021	3	20	0
0.000021	4	20	8
0.000021	5	20	14
0.000021	6	20	2
0.00007	1	20	2
0.00007	2	20	3
0.00007	3	20	3
0.00007	4	20	13

0.00007	5	20	11
0.00007	6	20	9
0.00021	1	20	8
0.00021	2	20	7
0.00021	3	20	3
0.00021	4	20	16
0.00021	5	20	12
0.00021	6	20	6
0.0007	1	20	13
0.0007	2	20	6
0.0007	3	20	10
0.0007	4	20	18
0.0007	5	20	14
0.0007	6	20	13
0.0021	1	20	12
0.0021	2	20	14
0.0021	3	20	14
0.0021	4	20	13
0.0021	5	20	15
0.0021	6	20	15

A. mellifera -Cerconil

Concentration (g/mL)	colony	total	dead
**control**	**1**	20	4
**control**	**2**	20	0
**control**	**3**	20	1
**control**	**4**	20	0
**control**	**5**	20	0
**control**	**6**	20	1
**0.00014**	**1**	20	3
**0.00014**	**2**	20	2
**0.00014**	**3**	20	6
**0.00014**	**4**	20	1
**0.00014**	**5**	20	1
**0.00014**	**6**	20	2
**0.00042**	**1**	20	8
**0.00042**	**2**	20	4
**0.00042**	**3**	20	5
**0.00042**	**4**	20	6
**0.00042**	**5**	20	3
**0.00042**	**6**	20	7
**0.0014**	**1**	20	15
**0.0014**	**2**	20	6
**0.0014**	**3**	21	8
**0.0014**	**4**	20	14
**0.0014**	**5**	20	5
**0.0014**	**6**	20	9

**0.0042**	**1**	20	15
**0.0042**	**2**	20	16
**0.0042**	**3**	20	18
**0.0042**	**4**	20	17
**0.0042**	**5**	20	16
**0.0042**	**6**	20	11
**0.014**	**1**	20	20
**0.014**	**2**	20	20
**0.014**	**3**	20	18
**0.014**	**4**	20	17
**0.014**	**5**	20	18
**0.014**	**6**	20	12

***P. helleri* -Cerconil**

Concentration (g/mL)	colony	total	dead
**control**	**1**	20	0
**control**	**2**	20	2
**control**	**3**	20	7
**control**	**4**	20	2
**control**	**5**	20	3
**control**	**6**	20	0
**0.0000014**	**1**	22	0
**0.0000014**	**2**	21	1
**0.0000014**	**3**	20	4
**0.0000014**	**4**	20	3
**0.0000014**	**5**	20	0
**0.0000014**	**6**	20	0
**0.0000028**	**1**	19	3
**0.0000028**	**2**	22	2
**0.0000028**	**3**	23	1
**0.0000028**	**4**	20	1
**0.0000028**	**5**	20	0
**0.0000028**	**6**	20	2
**0.000014**	**1**	20	1
**0.000014**	**2**	20	2
**0.000014**	**3**	20	3
**0.000014**	**4**	20	11
**0.000014**	**5**	20	0
**0.000014**	**6**	20	1
**0.00014**	**1**	19	6
**0.00014**	**2**	20	9
**0.00014**	**3**	21	0
**0.00014**	**4**	21	3
**0.00014**	**5**	20	0
**0.00014**	**6**	20	1
**0.00042**	**1**	20	11

**0.00042**	**2**	20	12
**0.00042**	**3**	20	11
**0.00042**	**4**	20	1
**0.00042**	**5**	20	0
**0.00042**	**6**	20	0
**0.0014**	**1**	20	2
**0.0014**	**2**	20	17
**0.0014**	**3**	20	14
**0.0014**	**4**	20	12
**0.0014**	**5**	20	10
**0.0014**	**6**	20	9

A. mellifera - Deltamethrin + Cerconil (0.01 g/mL)

**Deltamethrin concentration (g/mL)**	**colony**	**total**	**dead**
**control**	**1**	20	0
**control**	**2**	20	0
**control**	**3**	20	3
**control**	**4**	20	1
**control**	**5**	20	0
**control**	**6**	20	1
**0.025**	**1**	20	0
**0.025**	**2**	20	0
**0.025**	**3**	20	0
**0.025**	**4**	20	1
**0.025**	**5**	20	3
**0.025**	**6**	20	4
**0.075**	**1**	20	2
**0.075**	**2**	20	5
**0.075**	**3**	20	2
**0.075**	**4**	20	5
**0.075**	**5**	20	3
**0.075**	**6**	20	0
**0.125**	**1**	20	1
**0.125**	**2**	20	3
**0.125**	**3**	20	0
**0.125**	**4**	20	0
**0.125**	**5**	20	3
**0.125**	**6**	20	0
**0.175**	**1**	20	1
**0.175**	**2**	20	5
**0.175**	**3**	20	3
**0.175**	**4**	20	1
**0.175**	**5**	20	4
**0.175**	**6**	20	0
**0.25**	**1**	20	0
**0.25**	**2**	20	2

**0.25**	**3**	20	11
**0.25**	**4**	20	19
**0.25**	**5**	20	20
**0.25**	**6**	20	18
**0.75**	**1**	20	6
**0.75**	**2**	20	15
**0.75**	**3**	20	20
**0.75**	**4**	20	20
**0.75**	**5**	20	20
**0.75**	**6**	20	20
**1.25**	**1**	20	13
**1.25**	**2**	20	20
**1.25**	**3**	20	20
**1.25**	**4**	20	20
**1.25**	**5**	20	20
**1.25**	**6**	20	20
**1.75**	**1**	20	12
**1.75**	**2**	20	20
**1.75**	**3**	20	19
**1.75**	**4**	20	20
**1.75**	**5**	20	20
**1.75**	**6**	20	20
**2.25**	**1**	20	16
**2.25**	**2**	20	20
**2.25**	**3**	20	20
**2.25**	**4**	20	20
**2.25**	**5**	20	20
**2.25**	**6**	20	20

P. helleri - Deltamethrin + Cerconil (0.01 g/mL)

Deltamethrin concentration (g/mL)	colony	total	dead
**control**	**1**	21	1
**control**	**2**	19	0
**control**	**3**	19	2
**control**	**4**	20	0
**0.00000025**	**1**	21	8
**0.00000025**	**2**	20	2
**0.00000025**	**3**	19	15
**0.00000025**	**4**	19	6
**0.00000075**	**1**	20	8
**0.00000075**	**2**	21	4
**0.00000075**	**3**	20	1
**0.00000075**	**4**	20	6
**0.0000025**	**1**	20	1
**0.0000025**	**2**	20	4
**0.0000025**	**3**	20	10
**0.0000025**	**4**	20	7

**0.0000125**	**1**	20	10
**0.0000125**	**2**	19	1
**0.0000125**	**3**	20	0
**0.0000125**	**4**	20	7
**0.000025**	**1**	21	20
**0.000025**	**2**	19	13
**0.000025**	**3**	20	9
**0.000025**	**4**	20	20
**0.000075**	**1**	20	2
**0.000075**	**2**	20	3
**0.000075**	**3**	20	16
**0.000075**	**4**	19	5

A. mellifera **- Deltamethrin + thiophanate-methyl (0.01 g/mL)**

**Deltamethrin concentration (g/mL)**	**colony**	**total**	**dead**
**control**	**1**	20	1
**control**	**2**	20	0
**control**	**3**	20	1
**control**	**4**	20	3
**control**	**5**	20	0
**control**	**6**	20	0
**0.025**	**1**	20	2
**0.025**	**2**	20	1
**0.025**	**3**	20	0
**0.025**	**4**	20	0
**0.025**	**5**	20	6
**0.025**	**6**	20	2
**0.25**	**1**	20	8
**0.25**	**2**	20	8
**0.25**	**3**	20	5
**0.25**	**4**	20	4
**0.25**	**5**	20	7
**0.25**	**6**	20	5
**0.75**	**1**	20	20
**0.75**	**2**	20	20
**0.75**	**3**	20	11
**0.75**	**4**	20	15
**0.75**	**5**	20	20
**0.75**	**6**	20	20
**1.25**	**1**	20	20
**1.25**	**2**	20	20
**1.25**	**3**	20	18
**1.25**	**4**	20	20
**1.25**	**5**	20	20
**1.25**	**6**	20	20
**1.75**	**1**	20	20
**1.75**	**2**	20	20

**1.75**	**3**	20	20
**1.75**	**4**	20	20
**1.75**	**5**	20	20
**1.75**	**6**	20	20
**2.25**	**1**	16	16
**2.25**	**2**	20	20
**2.25**	**3**	20	20
**2.25**	**4**	20	20
**2.25**	**5**	20	20
**2.25**	**6**	20	20

P. helleri - Deltamethrin + thiophanate-methyl (0.01 g/mL)

Deltamethrin concentration (g/mL)	colony	total	dead
**control**	**1**	20	1
**control**	**2**	20	2
**control**	**3**	20	1
**0.000075**	**1**	20	20
**0.000075**	**2**	20	7
**0.000075**	**3**	20	1
**0.00025**	**1**	20	2
**0.00025**	**2**	20	1
**0.00025**	**3**	20	1
**0.0025**	**1**	20	7
**0.0025**	**2**	20	20
**0.0025**	**3**	20	7
**0.0125**	**1**	20	20
**0.0125**	**2**	20	20
**0.0125**	**3**	20	20
**0.025**	**1**	20	20
**0.025**	**2**	20	20
**0.025**	**3**	20	20
**0.075**	**1**	20	20
**0.075**	**2**	20	20
**0.075**	**3**	20	20
**0.075**	**1**	20	20
**0.075**	**2**	20	20
**0.075**	**3**	20	20

A. mellifera - Deltamethrin + Chlorothalonil (0.01 g/mL)

Deltamethrin concentration (g/mL)	colony	total	dead
**control**	**1**	20	0

**control**	**2**	20	0
**control**	**3**	20	2
**control**	**4**	20	2
**control**	**5**	20	0
**control**	**6**	20	0
**0.025**	**1**	20	2
**0.025**	**2**	20	1
**0.025**	**3**	20	1
**0.025**	**4**	20	0
**0.025**	**5**	20	4
**0.025**	**6**	20	12
**0.25**	**1**	20	11
**0.25**	**2**	20	5
**0.25**	**3**	20	8
**0.25**	**4**	20	11
**0.25**	**5**	20	14
**0.25**	**6**	20	19
**0.75**	**1**	20	20
**0.75**	**2**	20	9
**0.75**	**3**	20	19
**0.75**	**4**	20	20
**0.75**	**5**	20	20
**0.75**	**6**	20	19
**1.25**	**1**	20	20
**1.25**	**2**	20	20
**1.25**	**3**	20	20
**1.25**	**4**	20	20
**1.25**	**5**	20	20
**1.25**	**6**	20	20
**1.75**	**1**	20	20
**1.75**	**2**	20	20
**1.75**	**3**	20	20
**1.75**	**4**	20	20
**1.75**	**5**	20	20
**1.75**	**6**	20	20
**2.25**	**1**	20	20
**2.25**	**2**	20	20
**2.25**	**3**	20	20
**2.25**	**4**	20	20
**2.25**	**5**	20	20
**2.25**	**6**	20	20

P. helleri **- Deltamethrin+chlorothalonil (0.01 g/mL)**

Deltamethrin concentration (g/mL)	colony	total	dead
**control**	**1**	20	1
**control**	**2**	20	0

**control**	**3**	20	0
**0.0000025**	**1**	20	2
**0.0000025**	**2**	20	2
**0.0000025**	**3**	20	0
**0.000025**	**1**	20	1
**0.000025**	**2**	20	0
**0.000025**	**3**	20	0
**0.000025**	**1**	20	1
**0.000025**	**2**	20	1
**0.000025**	**3**	20	10
**0.000075**	**1**	20	2
**0.000075**	**2**	20	1
**0.000075**	**3**	20	2
**0.00025**	**1**	20	2
**0.00025**	**2**	20	1
**0.00025**	**3**	20	0
**0.0025**	**1**	20	11
**0.0025**	**2**	20	7
**0.0025**	**3**	20	7
**0.0125**	**1**	20	20
**0.0125**	**2**	20	20
**0.0125**	**3**	20	20

A. mellifera - imidacloprid+ Chlorothalonil (0.01 g/mL)

**imidacloprid concentration (g/mL)**	**colony**	**total**	**dead**
**control**	**1**	20	0
**control**	**2**	20	1
**control**	**3**	20	3
**control**	**4**	20	1
**0.00000021**	**1**	20	0
**0.00000021**	**2**	20	1
**0.00000021**	**3**	20	7
**0.00000021**	**4**	20	2
**0.0000021**	**1**	20	1
**0.0000021**	**2**	20	4
**0.0000021**	**3**	20	3
**0.0000021**	**4**	20	1
**0.000021**	**1**	20	1
**0.000021**	**2**	20	5
**0.000021**	**3**	20	8
**0.000021**	**4**	20	5
**0.00021**	**1**	20	13
**0.00021**	**2**	20	12
**0.00021**	**3**	20	14
**0.00021**	**4**	20	12
**0.0021**	**1**	20	20
**0.0021**	**2**	20	20

**0.0021**	**3**	20	19
**0.0021**	**4**	20	20

P. helleri - imidacloprid+ Chlorothalonil (0.01 g/mL)

**imidacloprid concentration (g/mL)**	**colony**	**total**	**dead**
**control**	**1**	20	2
**control**	**2**	20	0
**control**	**3**	20	2
**control**	**4**	20	3
**0.000000021**	**1**	20	13
**0.000000021**	**2**	20	4
**0.000000021**	**3**	20	10
**0.000000021**	**4**	20	12
**0.00000021**	**1**	20	6
**0.00000021**	**2**	20	6
**0.00000021**	**3**	20	6
**0.00000021**	**4**	20	6
**0.0000021**	**1**	20	10
**0.0000021**	**2**	20	3
**0.0000021**	**3**	20	9
**0.0000021**	**4**	20	10
**0.000021**	**1**	20	14
**0.000021**	**2**	20	13
**0.000021**	**3**	20	12
**0.000021**	**4**	20	10
**0.00021**	**1**	20	16
**0.00021**	**2**	20	19
**0.00021**	**3**	20	20
**0.00021**	**4**	20	12
